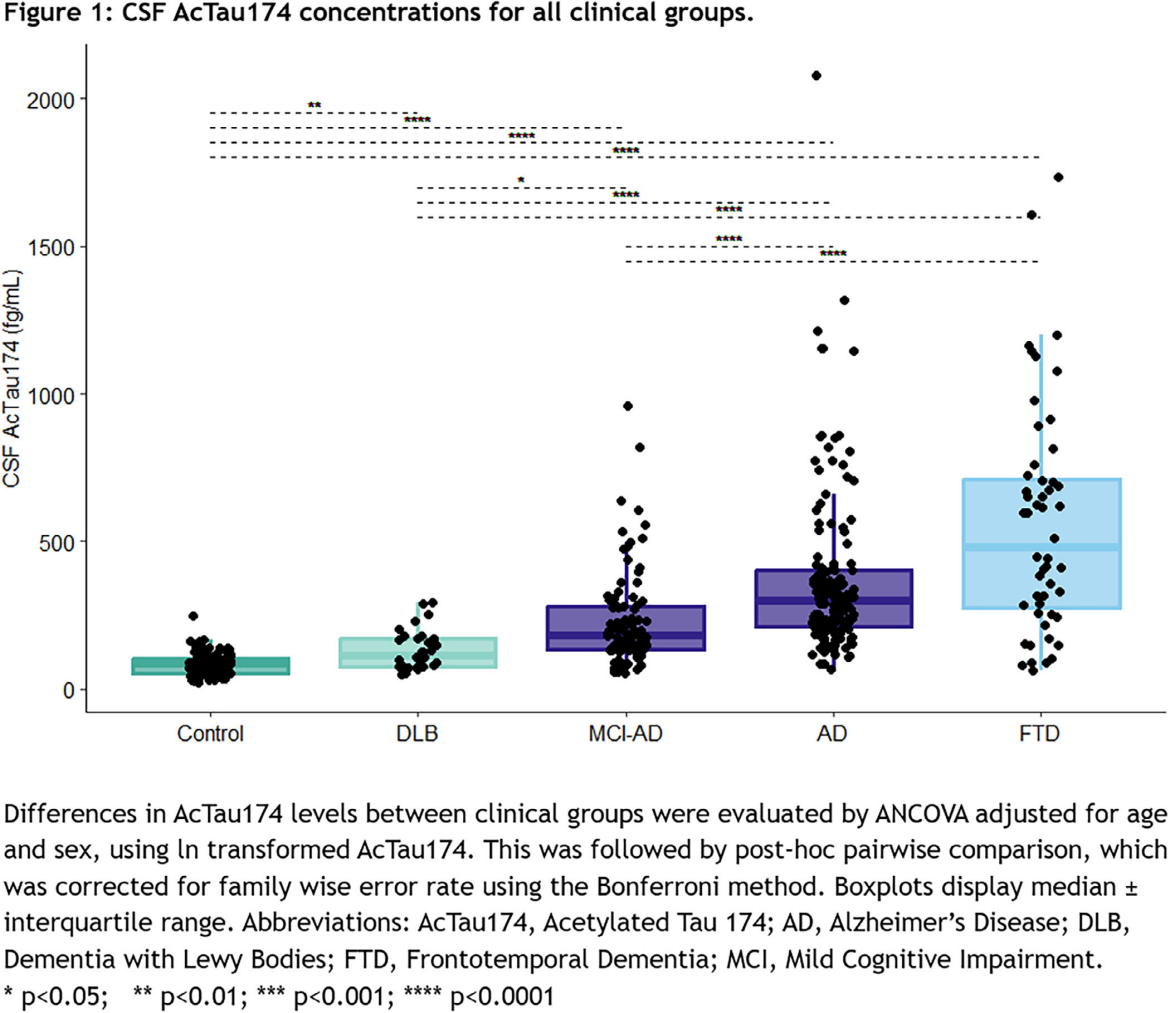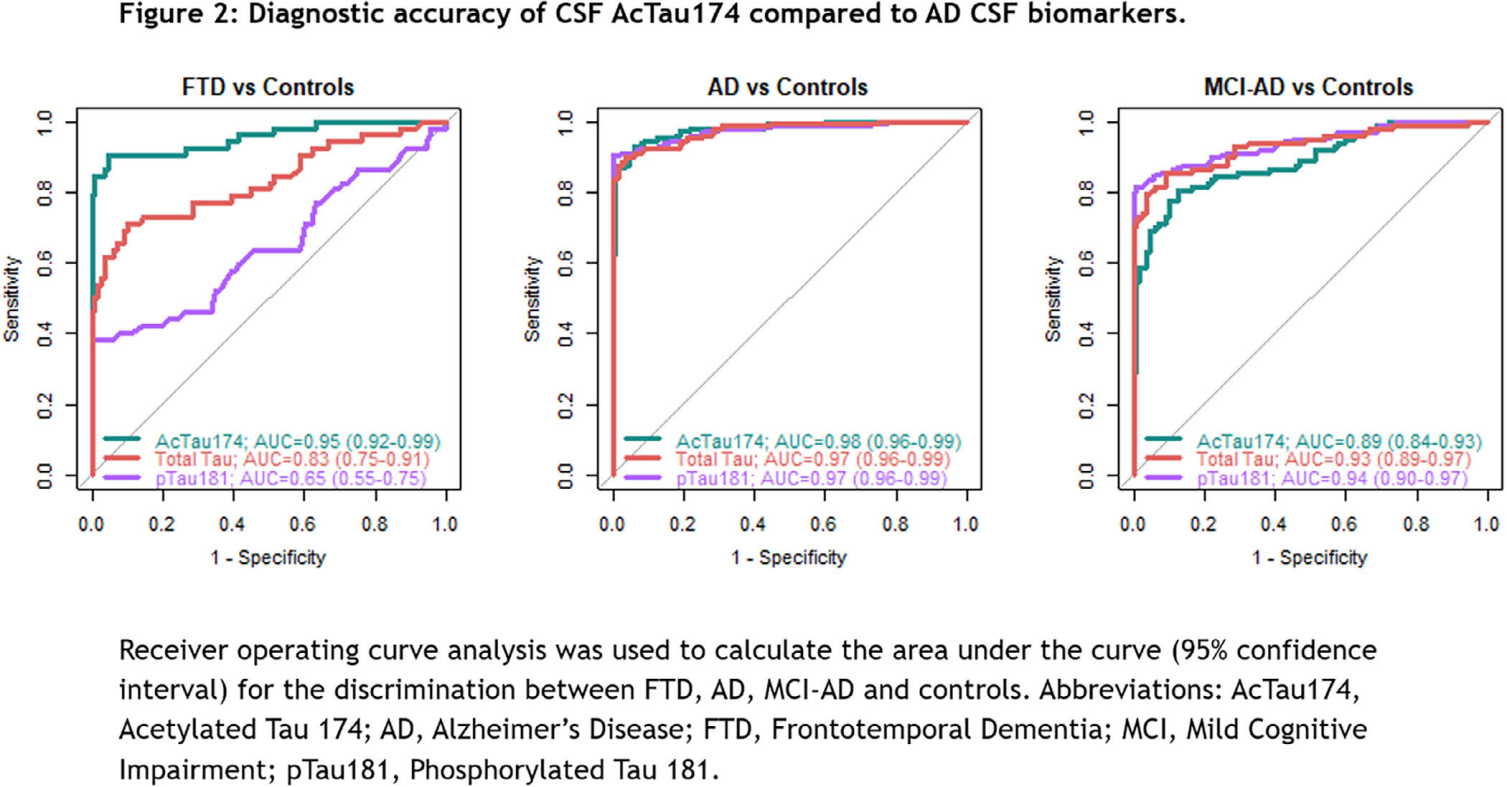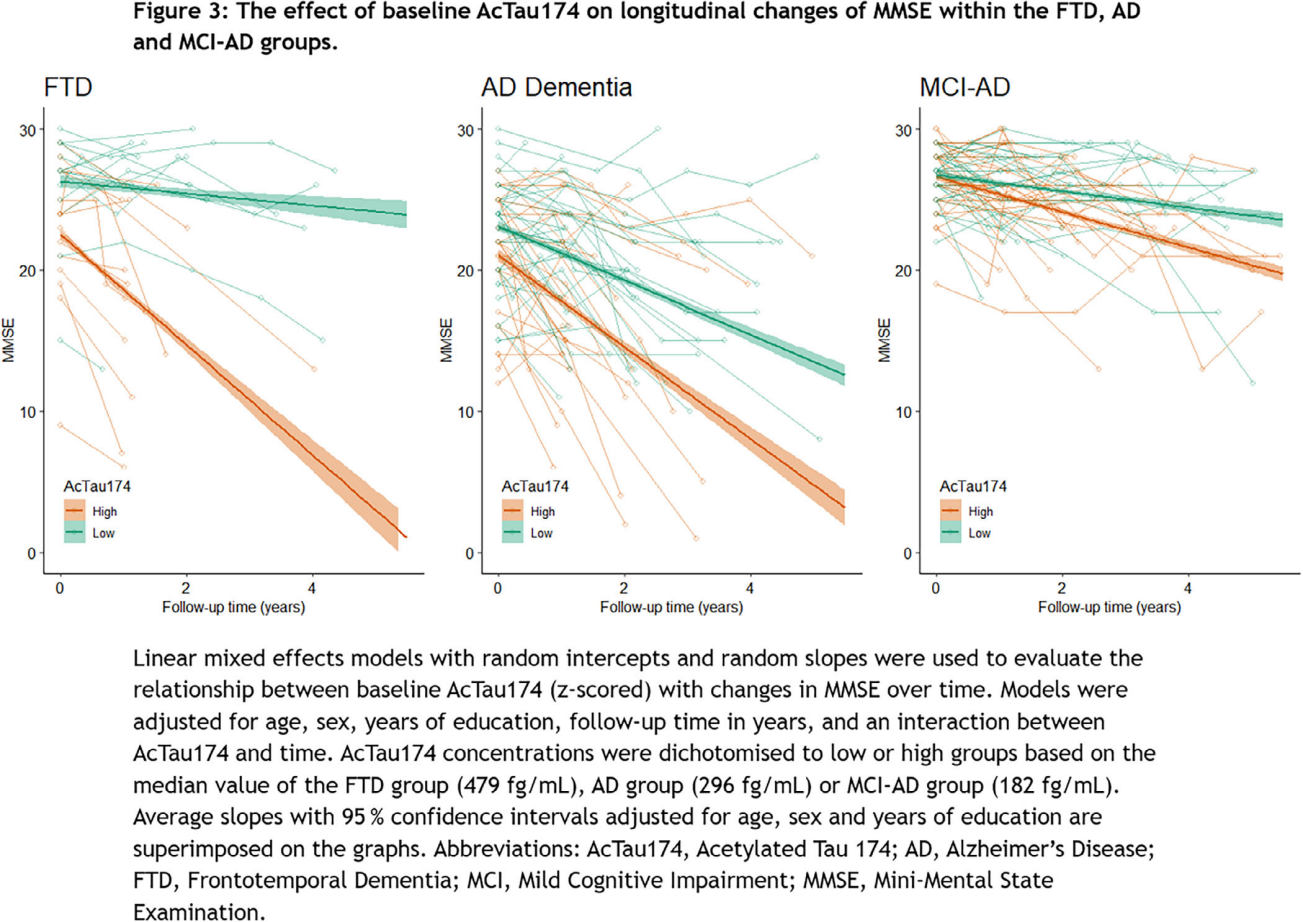# CSF Acetylated Tau‐174 as a novel diagnostic and prognostic biomarker in Frontotemporal Dementia and Alzheimer’s Disease

**DOI:** 10.1002/alz70861_108639

**Published:** 2025-12-23

**Authors:** Madison I. J. Honey, Yanaika S. Hok‐A‐Hin, Elisabeth H Thijssen, Babette van der Weijden, Erik Stoops, Sterre C.M. de Boer, Flora H. Duits, Emma L. van der Ende, Wiesje M. van der Flier, Inge M.W. Verberk, Yolande A.L. Pijnenburg, Li Gan, Charlotte E. Teunissen

**Affiliations:** ^1^ Neurochemistry Laboratory, Department of Laboratory Medicine, Amsterdam Neuroscience, Vrije Universiteit Amsterdam, Amsterdam UMC, Amsterdam Netherlands; ^2^ ADx NeuroSciences NV, Ghent Belgium; ^3^ Alzheimer Center Amsterdam, Department of Neurology, Amsterdam Neuroscience, Vrije Universiteit Amsterdam, Amsterdam UMC, Amsterdam Netherlands; ^4^ Alzheimer Center Amsterdam, Department of Neurology, Amsterdam UMC, location VUmc, Amsterdam Netherlands; ^5^ Helen and Robert Appel Alzheimer's Disease Research Institute, Brain and Mind Research Institute, Weill Cornell Medicine, New York, NY USA

## Abstract

**Background:**

Post‐translational modifications of tau, such as acetylation, are emerging as key mechanisms in tau pathology. Tau acetylation at lysine 174 (AcTau174) is elevated in primary neurons with frontotemporal dementia (FTD)‐linked mutations and in early Alzheimer’s disease (AD) brain tissue. However, the utility of AcTau174 as a cerebrospinal fluid (CSF) biomarker across dementias is unknown, including in FTD, where AD‐type tau CSF biomarkers are typically not elevated. We investigated the diagnostic and prognostic potential of AcTau174 in a well‐characterized clinical cohort.

**Method:**

Using our in‐house developed Simoa assay, we measured AcTau174 in CSF samples from the Amsterdam Dementia Cohort (109 non‐neurodegenerative controls, 52 FTD, 145 AD, 97 mild cognitive impairment due to AD (MCI‐AD), and 31 dementia with Lewy bodies (DLB)).

**Result:**

AcTau174 levels were elevated in all dementia groups compared to controls, with a stepwise increase from controls to MCI‐AD (2.2‐fold) to AD dementia (3.5‐fold, Figure 1). The strongest increase compared to controls was observed in the FTD group, wherein AcTau174 distinguished FTD from controls with a high area under the curve (AUC) accuracy of 0.95 (95%CI:0.92;0.99), significantly outperforming CSF pTau181 (AUC=0.65, 95%CI:0.55;0.75) and CSF Tau (AUC=0.83, 95%CI:0.75;0.91). AcTau174 showed high accuracy for differentiating AD (AUC=0.98, 95%CI:0.96;0.99) and MCI‐AD (AUC=0.89, 95%CI:0.84;0.93) from controls (Figure 2). In FTD, AcTau174 correlated with MMSE (ρ=‐0.29, *p* =0.039), and in AD with the frontal assessment battery score (ρ=‐0.35, *p* =0.0001). In FTD, higher baseline CSF AcTau174 was associated with a faster rate of decline in MMSE over time (β=‐1.64, 95%CI:‐2.61;‐0.87, *p* =0.0014, Figure 3), which was not observed for CSF pTau181 or Tau. Higher baseline CSF AcTau174 also predicted faster MMSE decline within AD (β=‐1.66, 95%CI:‐2.44;‐0.90, *p* <0.0001) and MCI‐AD (β=‐0.67, 95%CI:‐1.09;‐0.27, *p* =0.002, Figure 3).

**Conclusion:**

CSF AcTau174 shows great diagnostic value across neurodegenerative dementias and is strongly associated with clinical progression. In FTD, where trials are limited by clinical heterogeneity and lack of fluid biomarkers, AcTau174 may aid in identifying patients at risk of rapid decline, supporting trial enrichment and more targeted intervention strategies. Its predictive capacity for MMSE decline highlights its potential as a progression biomarker, both for patient monitoring and therapeutic development.